# Permethrin Versus Benzyl Benzoate for the Treatment of Scabies: A Systematic Review and Meta-Analysis of Randomized Controlled Trials

**DOI:** 10.7759/cureus.79295

**Published:** 2025-02-19

**Authors:** Nouf Alenezi, Reem AlQusaimi, Hanan Alajmi, Ebtehal Y AlMutairi, Abdulwahab T Alenezi, Abdulbadih R Saad, Nafisah Al Radhwan

**Affiliations:** 1 Medicine and Surgery, Kuwait Institute for Medical Specializations, Kuwait City, KWT; 2 Dentistry, Saad Al-Abdullah Health Center Block 2, Al Jahra, KWT; 3 Family and Community Medicine, Arabian Gulf University, Manama, BHR; 4 Obstetrics and Gynecology, Imam Abdulrahman Bin Faisal University, Dammam, SAU

**Keywords:** lesion severity, meta-analysis, scabies, topical benzyl benzoate, topical permethrin

## Abstract

Scabies is a common skin infestation with a high prevalence in populations with low socioeconomic conditions. The topical application of benzyl benzoate (BB) is effective due to its neurotoxic effects on mites; however, its efficacy remains inconsistent and ambiguous across studies. In contrast, topical permethrin 5% has emerged as one of the most widely prescribed treatments for scabies, attributed to its relatively high efficacy. Recently, several studies have evaluated the effectiveness of topical permethrin, highlighting the need for a comprehensive synthesis of evidence. Therefore, we conducted a systematic review and meta-analysis of randomized controlled trials (RCTs) to assess the efficacy of topical permethrin compared to BB in treating scabies. A systematic search of Scopus, PubMed, Web of Science (WOS), and Cochrane Central was performed from inception until January 2025 to identify RCTs comparing the effectiveness of topical permethrin and BB. The primary outcomes were clinical cure rates of scabies lesions and pruritus. Risk ratios (RRs) with 95% confidence intervals (CIs) were calculated using a random-effects model for dichotomous data. All statistical analyses were conducted using STATA 18MP (StataCorp LLC, College Station, USA). A total of seven RCTs, including 783 patients, met the inclusion criteria. Topical permethrin demonstrated significantly higher clinical cure rates for both scabies lesions and pruritus in the first week of treatment, with improvements of 30% and 23%, respectively (RR = 1.30; 95% CI: 1.11-1.53; p < 0.001 for scabies lesions, and RR = 1.23; 95% CI: 1.04-1.47; p = 0.02 for pruritus), compared to BB. However, no significant differences were observed between the two treatments in subsequent follow-up assessments. In conclusion, topical permethrin provides a superior and faster improvement in the treatment of scabies compared to BB within the first week of therapy, with no significant difference in later assessment durations, highlighting the superiority of permethrin is time-dependent. Further high-quality RCTs with long-term follow-up are warranted to confirm these findings and evaluate sustained efficacy.

## Introduction and background

Scabies is an infectious, severe itching skin disease that is caused by the human parasitic mite *Sarcoptes scabiei *infestation. The mite burrows into the top layer of the skin and lays its eggs, causing severe itching, especially at night [1]. The mite is transmitted through skin-to-skin contact, and with the asymptomatic infestation period, transmission may occur before the person develops symptoms [1]. Scabies affects 200 million people worldwide at any given time and 400 million as the total affected individuals each year [2]. Although it is more common in developing countries, recent years have shown an increasing incidence in high-income regions [2]. In 2009, the World Health Organization (WHO) declared scabies a neglected skin disease and a significant health problem for developing countries [3]. Scabies is not only a skin disease; it is associated with reduced ability to concentrate, sleep disturbances, social segmentation, and high costs on healthcare systems [4]. Patients with scabies can develop impetigo from scratching due to *Streptococcus pyogenes* or *Staphylococcus aureus* infection, which can lead to septicemia, rheumatic heart disease, and glomerulonephritis [5].

Various treatment options for scabies are topical, such as permethrin 5% or benzyl benzoate (BB) 10-25%, and oral, such as ivermectin. Permethrin 5% cream is widely used; it is applied once a week for two weeks before bed to allow skin contact of 8 to 10 hours [3]. Although it is effective and easily metabolized, it has recently been associated with scabies resistance, poor compliance among patients, and some allergic reactions [3]. BB 10-25% is applied three times a day with a skin contact time of almost 24 hours; it causes neurotoxicity in the mites. Due to its low cost, it is considered the drug of choice for low-income countries [6].

Several RCTs were conducted to compare different antiscabietic treatment options, including permethrin 5% and BB 10-25% [7-13]. With the conflict between the published results and the lack of conclusive collective evidence, we sought to conduct this systematic review and meta-analysis comparing permethrin 5% and BB 10-25%.

## Review

Methods

We performed this systematic review and meta-analysis adhering to the Preferred Reporting Items for Systematic Reviews and Meta-Analyses (PRISMA) guidelines [14]. We followed the methodologies proposed in the Cochrane Handbook for systematic reviews and meta-analyses of interventions [15].

Search Strategy and Data Sources

We conducted a comprehensive search on PubMed, Web of Science (WOS), Scopus, and Cochrane Central from inception until January 2025, using the following search terms: (scabies OR "seven-year itch" OR "Sarcoptes scabiei") AND (permethrin OR "permethrin 5%" OR nix OR "nix permethrin" OR elimite OR lyclear) AND (“benzyl benzoate” OR "benzyl benzoate 25%" OR "Phenylmethyl benzoate" OR "benzoic acid phenylmethyl ester" OR "BB Lotion" OR "Benzyl Ester of Benzoic Acid" OR Ascabin OR Ascabiol OR Ascarbin). Detailed search terms according to each database are summarized in Table 1. We retrieved only English studies published on humans. Additionally, we retrieved the references from included studies to ensure all relevant papers were included.

**Table 1:** Detailed search strategy for each database.

Database	Search strategy	Filter	Results
PubMed	((scabies[Title/Abstract] OR "seven-year itch"[Title/Abstract] OR "Sarcoptes scabiei"[MeSH] OR "Sarcoptes scabiei"[Title/Abstract]) AND (permethrin[Title/Abstract] OR "permethrin 5%"[Title/Abstract] OR nix[Title/Abstract] OR "nix permethrin"[Title/Abstract] OR elimite[Title/Abstract] OR lyclear[Title/Abstract]) AND ("benzyl benzoate"[Title/Abstract] OR "benzyl benzoate 25%"[Title/Abstract] OR "Phenylmethyl benzoate"[Title/Abstract] OR "benzoic acid phenylmethyl ester"[Title/Abstract] OR "BB Lotion"[Title/Abstract] OR "Benzyl Ester of Benzoic Acid"[Title/Abstract] OR Ascabin[Title/Abstract] OR Ascabiol[Title/Abstract] OR Ascarbin[Title/Abstract] OR Tenutex[Title/Abstract]))	All Fields	63
CENTRAL	(scabies OR "seven-year itch" OR "Sarcoptes scabiei") AND (permethrin OR "permethrin 5%" OR nix OR "nix permethrin" OR elimite OR lyclear) AND ("benzyl benzoate" OR "benzyl benzoate 25%" OR "Phenylmethyl benzoate" OR "benzoic acid phenylmethyl ester" OR "BB Lotion" OR "Benzyl Ester of Benzoic Acid" OR Ascabin OR Ascabiol OR Ascarbin OR Tenutex)	Title Abstract Keyword	17
Web of Science	TITLE-ABS-KEY (scabies OR "seven-year itch" OR "Sarcoptes scabiei") AND TITLE-ABS-KEY (permethrin OR "permethrin 5%" OR nix OR "nix permethrin" OR elimite OR lyclear) AND TITLE-ABS-KEY ("benzyl benzoate" OR "benzyl benzoate 25%" OR "Phenylmethyl benzoate" OR "benzoic acid phenylmethyl ester" OR "BB Lotion" OR "Benzyl Ester of Benzoic Acid" OR Ascabin OR Ascabiol OR Ascarbin OR Tenutex)	All Fields	75
Scopus	TS=(scabies OR "seven-year itch" OR "Sarcoptes scabiei") AND TS=(permethrin OR "permethrin 5%" OR nix OR "nix permethrin" OR elimite OR lyclear) AND TS=("benzyl benzoate" OR "benzyl benzoate 25%" OR "Phenylmethyl benzoate" OR "benzoic acid phenylmethyl ester" OR "BB Lotion" OR "Benzyl Ester of Benzoic Acid" OR Ascabin OR Ascabiol OR Ascarbin OR Tenutex)	Article title, Abstract, Keywords	313

Eligibility Criteria and Study Selection

We screened the search results by following a two-step approach. Title and abstract screening were the first steps, followed by full-text screening of the eligible studies, by which we identified all RCTs that met the study inclusion criteria.

We included only randomized controlled trials (RCTs) that met the following population, intervention, comparison, and outcome (PICO) criteria: P) patients with scabies; I) intervention: topical permethrin 5%; C) comparator: topical BB 10-25%; and O) reported outcomes of interest in an intention-to-treat analysis. We excluded studies not comparing topical permethrin or topical BB, unpublished data, conference abstracts, and observational studies.

Endpoints

The primary outcomes of interest were the cumulative clinical cure rate from scabies lesions and pruritus assessed at the first, second, fourth, and sixth weeks post-treatment using a numerical grading scale or the visual analog scale (VAS). The numerical grading scale ranged from 0, representing the lowest score, to 10, representing the highest score. Additionally, the VAS score is a 10-point scale with “10” indicating the worst pain ever and “0” indicating no pain at all.

Quality Assessment and Data Extraction

Risk of bias assessment was conducted by two independent authors for all the included studies using the Cochrane Risk of bias 2 tool (ROB2) for evaluating RCTs [16]. Five different domains of ROB2 account for different methodologies of bias, which include reporting bias (selection of the reported outcomes), selection bias (randomization process), attrition bias (missing outcome data), detection bias (outcome measurements), and performance bias (deviation from intended interventions). The decisions were labeled as “low risk of bias,” “some concerns,” and “high risk of bias.” Another discussion with an additional author was warranted if there were any disagreements between the two authors.

A standardized Excel sheet was used to extract data from the included studies. The data extracted included four domains: (1) a summary of the included studies; (2) characteristics of the included patients; (3) risk of bias domains; and (4) measurements of the studied outcomes.

Statistical Analysis

Dichotomous data were extracted as the frequency of events and the total number of patients included at each follow-up point from each study. For each outcome, the pooled risk ratio (RR) with its 95% confidence interval (CI) was calculated using the DerSimonian-Laird random-effects model. Heterogeneity was assessed using the Cochrane Q test, and the I² measure was determined across all studies. A p-value less than 0.05 and an I² value ≥50% were deemed significant heterogeneity among the included studies. In case of significant heterogeneity, a leave-one-out sensitivity analysis was performed to explore the potential sources of the proposed heterogeneity, and the Galbraith plot was used to visualize studies outside the 95% precision area. All statistical analyses were performed on STATA 18MP (StataCorp LLC, College Station, USA) using the packages “meta esize” and “meta forest plot” to pool the effect estimate and the corresponding 95% CI.

Results

Search Results

**Figure 1:**
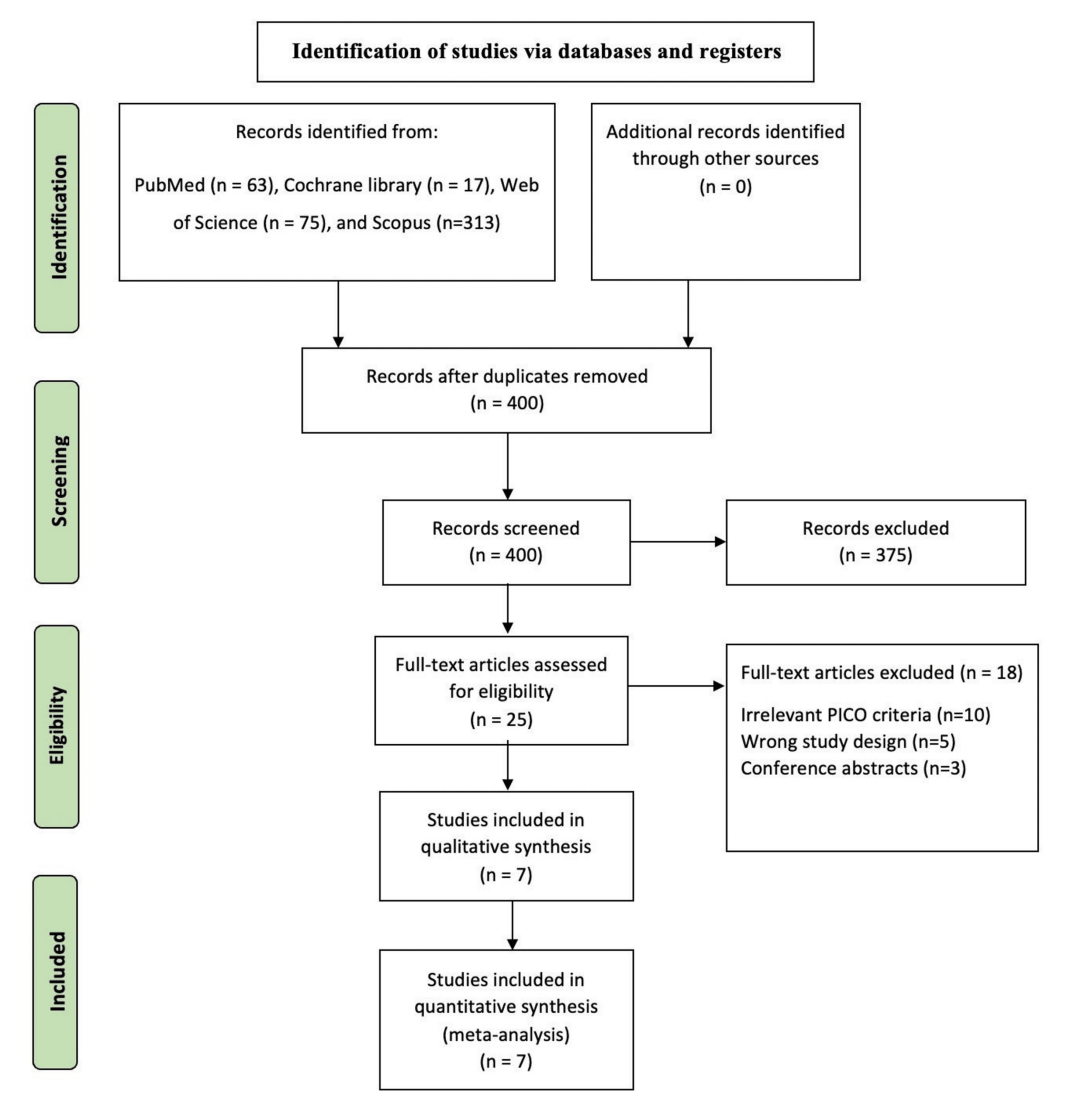
PRISMA flow diagram of the selection process. PRISMA: Preferred Reporting Items for Systematic Reviews and Meta-Analyses; PICO: population, intervention, comparison, and outcome

We identified 468 citations, of which 454 were excluded after title, abstract, and full-text screening according to the pre-specified PICO. Finally, seven RCTs [7-13] were included in the final meta-analysis. The PRISMA diagram of the study selection process is shown in Figure 1.

Studies Characteristics and Risk of Bias

Seven RCTs, including 783 patients, were included in the final analysis; 390 patients (49.8%) were treated with permethrin, and 393 (50.2%) were treated with BB. The summary and baseline characteristics of the included patients are summarized in Tables 2-3.

**Table 2:** Summary data of the included studies. References: [7-13]

Study ID	Study design	Country	Trial duration	Sample size	Trial arm		Patient criteria
					Intervention	Control	
Babu et al. 2019 A [7]	Randomized controlled trial	India	February 2016-February2018	N = 178	Permethrin (5%) cream, single application	Benzyl benzoate (10 to 25%) emulsion, three times	Patients with scabies
Babu et al. 2019 B [9]	Randomized controlled trial	India	February 2016-December 2017	N = 130	Permethrin (5%) cream, single application	Benzyl benzoate (25%) lotion, single application	Patients with scabies
Bachewar et al. 2009 [11]	Randomized controlled trial	India	March 2007-July 2007	N = 69	Permethrin (5%) cream, single application	Benzyl benzoate (25%) lotion, twice	Patients with scabies
Chitra et al. 2020 [13]	Randomized controlled trial	India	August 2013-July 2014	N = 100	Permethrin (5%) cream, single application	Benzyl benzoate (25%) lotion, single application	Patients with scabies
Manjhi et al. 2014 [8]	Randomized controlled trial	India	April 2011-March 2012	N = 120	Permethrin (5%) cream, single application	Benzyl benzoate (25%) lotion, single application	Patients with scabies
Meyersburg et al. 2024 [12]	Randomized controlled trial	Austria	September 2022-June 2023	N = 106	Permethrin (5%) cream, three times	Benzyl benzoate (25%) emulsion, three times	Patients with scabies
Mushtaq et al. 2009 [10]	Randomized controlled trial	Pakistan	January 2009-October 2009	N = 80	Permethrin (5%) cream, single application	Benzyl benzoate (25%) lotion, three times	Patients with scabies

**Table 3:** Baseline data of the included patients. References: [7-13]

Study ID	Group	Sample size	Age (years)	Sex	Endpoints assessment tools	Follow-up
				(Male/female)		
Babu et al. 2019 A [7]	Permethrin	n = 89	18.18 ± 11.33	Both genders	Numerical rating scale	Six weeks
	Benzyl benzoate	n = 89	28.86 ± 12.39	Both genders		
Babu et al. 2019 B [9]	Permethrin	n = 65	29.85 ± 8.66	Both genders	Visual analog scale (VAS)	Six weeks
	Benzyl benzoate	n = 65	27.12 ± 10.28	Both genders		
Bachewar et al. 2009 [11]	Permethrin	n = 34	(12-41) range	Not reported	Visual analog scale (VAS)	Two weeks
	Benzyl benzoate	n = 35	(12-41) range	Not reported		
Chitra et al. 2020 [13]	Permethrin	n = 50	(11-31) range	Both genders	Numerical rating scale	Four weeks
	Benzyl benzoate	n = 50	(11-31) range	Both genders		
Manjhi et al. 2014 [8]	Permethrin	n = 60	(5-60) range	Both genders	Visual analog scale (VAS)	Six weeks
	Benzyl benzoate	n = 60	(5-60) range	Both genders		
Meyersburg et al. 2024 [12]	Permethrin	n = 52	25.5 ± 11.2	Both genders	Numerical rating scale	Three weeks
	Benzyl benzoate	n = 54	30.7 ± 18.5	Both genders		
Mushtaq et al. 2009 [10]	Permethrin	n = 40	(10-35) range	Both genders	Visual analog scale (VAS)	Two weeks
	Benzyl benzoate	n = 40	(10-35) range	Both genders		

**Figure 2:**
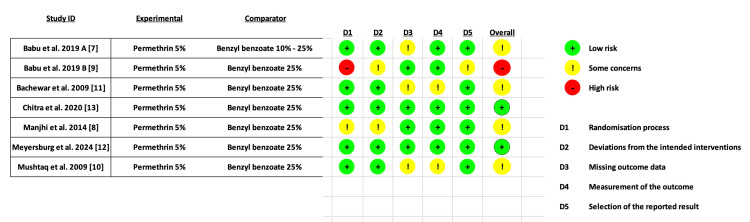
Risk of bias summary (ROB2 tool). References: [7-13] ROB: risk of bias 2

Only two studies had a low risk of bias, while most studies (n = 4 RCTs) had some concerns, mainly due to the missing outcome data domain, as shown in Figure 2.

Outcomes

**Figure 3:**
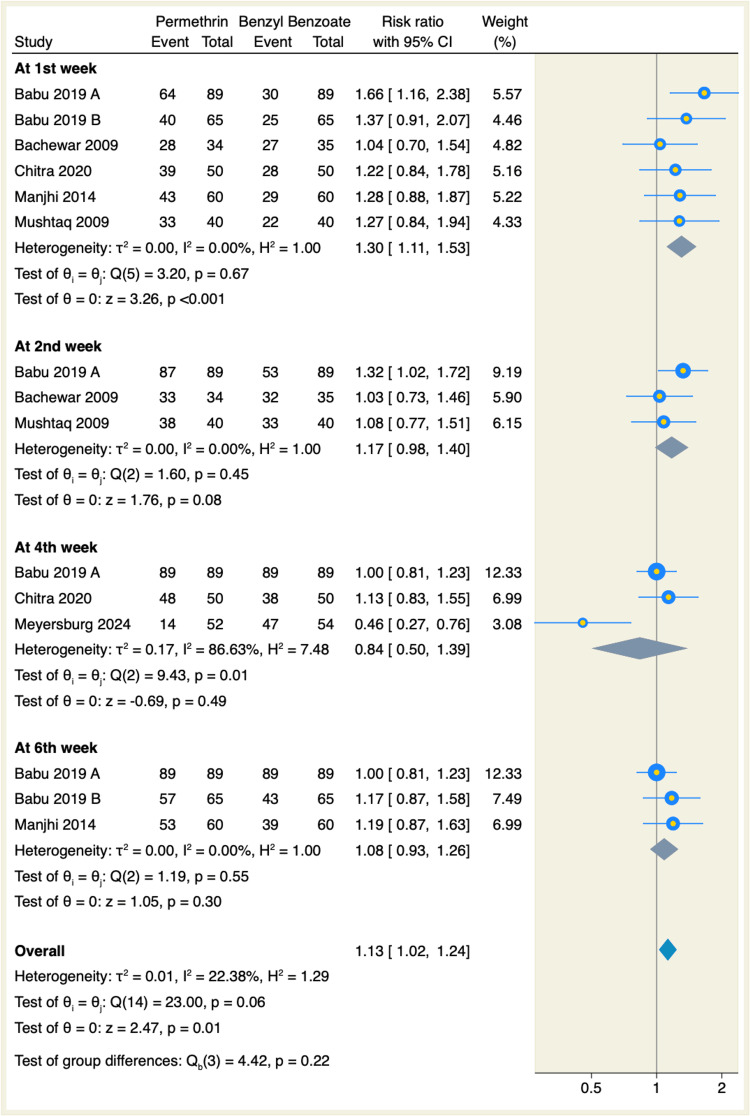
Forest plot of clinical cure rate from scabies lesions. References: [7-13]

All seven studies assessed the clinical cure rate from scabies lesions, of which topical permethrin significantly showed better cure rates with a 73% cure rate, compared to a 47.5% cure rate for topical BB in the first week, respectively. Moreover, the pooled RR from six RCTs favored topical permethrin (RR: 1.3, 95% CI: 1.11-1.53, p < 0.001; I² = 0.00, p = 0.67; number needed to treat (NNT) = 3.9 patients), as shown in <0.001; I² = 0.00, p = 0.67; NNT = 3.9 patients), as shown in Figure 3. On the other hand, there was no significant difference in the clinical cure rates from scabies lesions between topical permethrin and BB at the second week of follow-up (RR: 1.17, 95% CI: 0.98-1.4, p = 0.08), at the fourth week of follow-up (RR: 0.84, 95% CI: 0.5-1.39, p = 0.49), or at the sixth week of follow-up (RR: 1.08, 95% CI: 0.93-1.26, p = 0.3), as shown in Figure 3.

**Figure 4:**
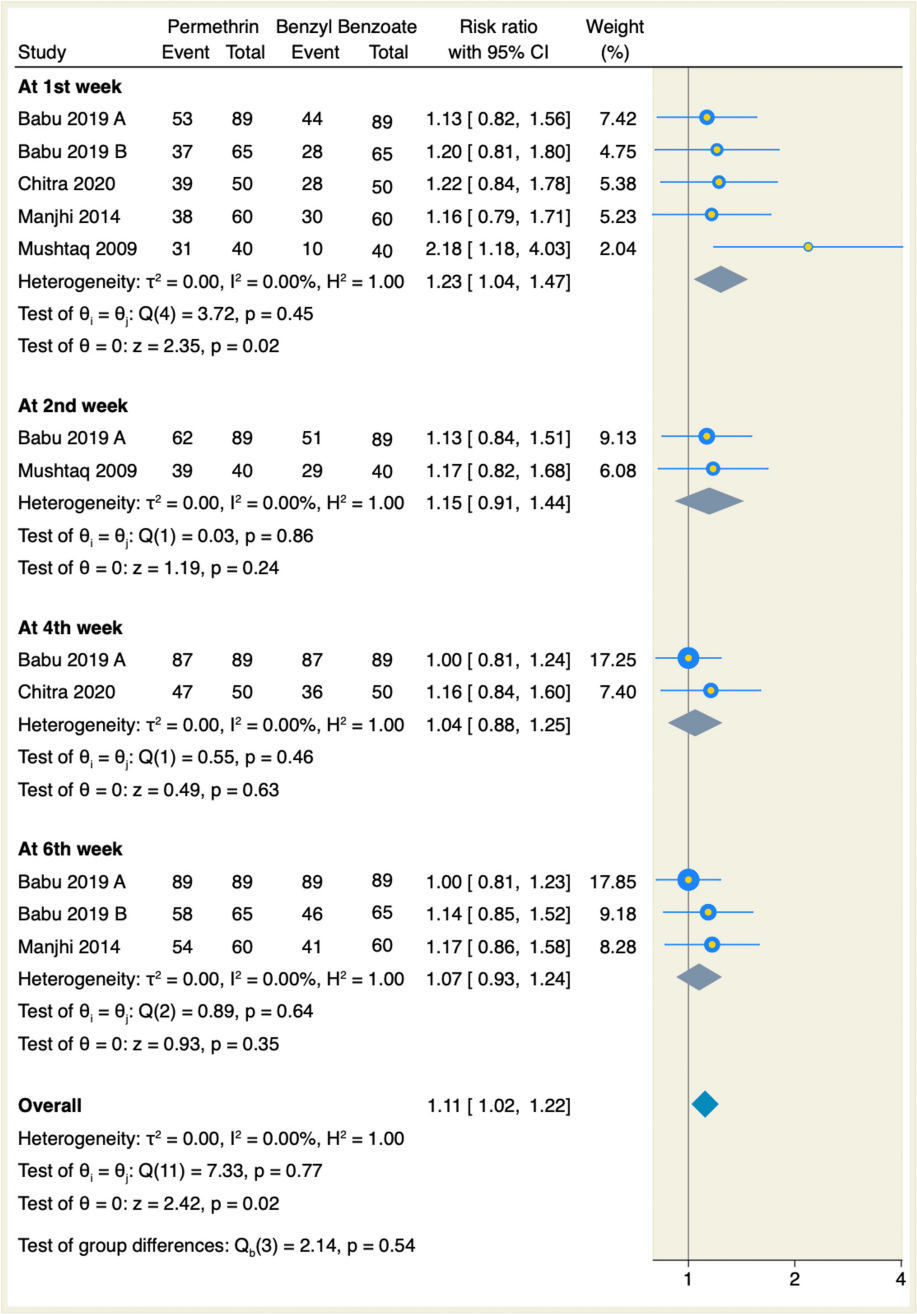
Forest plot of clinical cure rate from pruritis. References: [7-13]

Additionally, six studies reported the clinical cure rates from pruritus, of which topical permethrin significantly showed better cure rates with 65.1% compared to 46.1% for topical BB in the first week, respectively. Moreover, the pooled RR from five RCTs favored topical permethrin (RR: 1.23, 95% CI: 1.04-1.47, p = 0.02; I² = 0.00, p = 0.45), as shown in Figure 4. There were no significant differences in the other studied follow-up durations between topical permethrin and topical BB, as shown in Figure 4.

Discussion

This systematic review and meta-analysis is the largest study to date to investigate the comparative effectiveness of the topical administration of permethrin 5% and BB, including only published RCTs comprising 783 patients with scabies. Our meta-analysis showed that the administration of topical permethrin 5% was superior in terms of the cumulative cure rates, from lesion severity and pruritus, compared to the application of BB at the first week post-administration. However, no significant differences were noted at later stages of follow-up.

Treatment of scabies as a skin infection is not only for the anti-scabies effect but also for the relief from symptoms and associated secondary bacterial infections on top of the scabies infection. These secondary infections are primarily due to the disintegration in the continuity of the dermal layer caused by intensive itching [17]. Not only do scabies patients suffer from the symptoms related to their infection, but they also experience common adverse events caused by the exoskeletal remnants after the administration of anti-scabies drugs. This dead debris can cause dermal itching for a couple of weeks, even after successful treatment with an anti-scabies drug; thus, proper instructions to avoid the extensive use of anti-scabies drugs are mandatory [18].

In our study, we found that the cure rate from scabies lesions was 73% for topical permethrin and 47.5% for topical BB in the first week of assessment. Some key factors determine the cumulative cure rate, or, on the other hand, the rates of treatment failure. Among them, relapse and re-infestation are the most common causes of treatment failure, since the application of anti-scabies drugs requires adequate quantity and appropriate manner, as previously prescribed, such as applying the cream only to the affected parts of the body. Another common cause that leads to higher rates of treatment failure is the physician’s inability to provide proper instructions to patients, such as avoiding diluting or emulsifying the cream or missing the guided information to family members or contacts regarding the application of the cream as well [19].

The current findings are aligned with previous results from a recent network meta-analysis of different anti-scabies drugs, which concluded that the topical administration of permethrin was better than topical BB at 1-2 weeks [20].

Another large meta-analysis on the rates of scabies treatment failure, defined as re-infestation, recurrence of scabies, or lack of scabies susceptibility, found that the failure rate in the topical permethrin 5% group from 57 published studies, including 5142 patients, was 10.8% (95% CI: 7.5-14.5), with higher failure rates in the Eastern Mediterranean and Europe compared to Southeast Asia and America. Additionally, the treatment failure rates in the topical BB group from 34 published studies, including 6638 patients, were 25.3% (16.4-35.3), with higher failure rates in America and Africa compared to the Eastern Mediterranean, Southeast Asia, and Europe [21]. They also found that overall treatment failure was more likely to increase in recent studies compared to studies published before 2011. As for meta-regression, the year of publication was found to be a significant predictor of treatment failure, with a 0.27% (p = 0.017) increase for every incremental year of publication. Additionally, a similar trend was observed in topical permethrin application, where the failure rates increased by 0.58% per additional publication year [21].

The treatment failure rates or lower cure rates correlated with the dramatic increase in the incidence of scabies in recent years [21]. Also, possible resistance mechanisms are well-known for the most commonly used anti-scabies drugs [22]. Additionally, subtle changes in the formulation of drugs cannot be ruled out, which can lead to less effective treatment [23]. Moreover, information on the patients’ compliance is indeed important, as there is not enough data on the ease of administration of these drugs, which can, in turn, affect the compliance of the affected patients. The ease of administration depends on the mode of treatment application, whether oral route, lotion, cream, or topical appointment. Also, repeated applications of topical drugs should be given to eradicate the mites emerging from eggs. Furthermore, concurrent treatment of family members and physically contacted individuals, regardless of their symptoms, should also be adopted [24].

On the other hand, there is a need for a rational approach to treatment success for scabies, as many factors can contribute to higher cure rates, such as literacy, personal hygiene, level of socioeconomic status, and the existence of co-infection. Therefore, correct medications, correct dosing, proper treatment duration, and consideration of contacts can reduce the disease burden and alleviate its failure rates.

Our study had some limitations that need to be addressed in further evaluations. First, the included studies had relatively small sample sizes, leading to less precise effects. However, we pooled the data from all published RCTs to ensure adequate estimates. Additionally, numerous studies had a high risk of bias, and their participants were shown to be a source of diversity. Furthermore, most studies did not include patients with crusted scabies; thus, the applicability of our findings is limited to studies involving scabies patients. Finally, the included studies assessed the outcomes at different follow-up durations, and varying follow-up times may lead to biased results. We recommend further large-scale, multi-center RCTs with long-term follow-up to evaluate sustained efficacy.

## Conclusions

This systematic review and meta-analysis of seven RCTs demonstrated that the topical administration of permethrin showed better clinical cure rates in the first week compared to the application of topical BB. However, no significant differences were observed at later stages. Further large-scale, multi-center RCTs with long-term follow-up are needed to evaluate sustained efficacy.
